# From the stomach to locus coeruleus: new neural substrate for ghrelin’s effects on ingestive, motivated and anxiety-like behaviors

**DOI:** 10.3389/fphar.2023.1286805

**Published:** 2023-11-13

**Authors:** Ivana Maric, Lorena López-Ferreras, Yashaswini Bhat, Mohammed Asker, Stina Börchers, Lauren Bellfy, Suyeun Byun, Janine L. Kwapis, Karolina P. Skibicka

**Affiliations:** ^1^ Institute for Neuroscience and Physiology, University of Gothenburg, Gothenburg, Sweden; ^2^ Department of Nutritional Sciences, Pennsylvania State University, State College, PA, United States; ^3^ Department of Biology, Pennsylvania State University, State College, PA, United States; ^4^ Huck Institutes of the Life Sciences, Pennsylvania State University, State College, PA, United States

**Keywords:** ghrelin, hindbrain, locus coeruleus (LC), food motivation, anxiety-like behavior, LEAP2

## Abstract

Ghrelin, a stomach-derived orexigenic hormone, has a well-established role in energy homeostasis, food reward, and emotionality. Noradrenergic neurons of the locus coeruleus (LC) are known to play an important role in arousal, emotion, cognition, but recently have also been implicated in control of feeding behavior. Ghrelin receptors (the growth hormone secretagogue receptor, GHSR) may be found in the LC, but the behavioral effects of ghrelin signaling in this area are still unexplored. Here, we first determined whether GHSR are present in the rat LC, and demonstrate that GHSR are expressed on noradrenergic neurons in both sexes. We next investigated whether ghrelin controls ingestive and motivated behaviors as well as anxiety-like behavior by acting in the LC. To pursue this idea, we examined the effects of LC GHSR stimulation and blockade on food intake, operant responding for a palatable food reward and, anxiety-like behavior in the open field (OF) and acoustic startle response (ASR) tests in male and female rats. Our results demonstrate that intra-LC ghrelin administration increases chow intake and motivated behavior for sucrose in both sexes. Additionally, females, but not males, exhibited a potent anxiolytic response in the ASR. In order to determine whether activation of GHSR in the LC was necessary for feeding and anxiety behavior control, we utilized liver-expressed antimicrobial peptide 2 (LEAP2), a newly identified endogenous GHSR antagonist. LEAP2 delivered specifically into the LC was sufficient to reduce fasting-induced chow hyperphagia in both sexes, but food reward only in females. Moreover, blockade of GHSR in the LC increased anxiety-like behavior measured in the ASR test in both sexes. Taken together, these results indicate that ghrelin acts in the LC to alter ingestive, motivated and anxiety-like behaviors, with a degree of sex divergence.

## 1 Introduction

The orexigenic peptide ghrelin is mainly produced by the stomach and acts on the brain to promote feeding behavior (Wren et al., 2000). Ghrelin acts via the growth hormone secretagogue receptor (GHSR), which is abundantly expressed in brain regions known to regulate homeostatic and hedonic feeding (Kojima et al., 1999). For example, the receptor is found in the hypothalamus and brainstem areas, as well as areas within the mesolimbic pathway (Zigman et al., 2006). Peripherally administered ghrelin has consistently been shown to increase appetite in satiated rodents and humans, and ghrelin injections into discrete regions such as the arcuate nucleus (Arc), lateral hypothalamus (LH), ventral tegmental area (VTA) or the nucleus of the solitary tract (NTS) are potently orexigenic (Tschop et al., 2000; Abizaid et al., 2006; Faulconbridge et al., 2008; Egecioglu et al., 2010; Skibicka and Dickson, 2011; López-Ferreras et al., 2017; Hyland et al., 2020; Barrile et al., 2023; Wald et al., 2023). Furthermore, ghrelin signaling at the level of the mesolimbic system enhances the motivation for food as well as substances of abuse (Jerlhag et al., 2009; Skibicka et al., 2011; You et al., 2022a; You et al., 2022b). Thus, ghrelin has a well-established role in maintaining energy homeostasis and driving motivated behaviors beyond nutritional needs (Andrews, 2011; Skibicka and Dickson, 2011; Menzies et al., 2013).

Ghrelin release is stimulated by metabolic stress and negative energy status, however psychological stressors such as restraint and social defeat have been demonstrated to also trigger ghrelin secretion (Asakawa et al., 2001; Cummings et al., 2001; Kristenssson et al., 2006; Patterson et al., 2013; McKay et al., 2021). Ghrelin is therefore suggested to be the interface between metabolic disorders and stress response-related mood disorders such as anxiety and depression (Chuang and Zigman, 2010). In line with this, calorie restriction and exogenous ghrelin injections have both been shown to be anxiolytic (Lutter et al., 2008; Alvarez-Crespo et al., 2012; Toufexis et al., 2016; Borchers et al., 2022a). Although some contradictory reports indicate an anxiogenic effect following ghrelin administration (Asakawa et al., 2001; Carlini et al., 2002), implying that there might be a bidirectional effect depending on experimental conditions, namely, the access to food, it is clear that the ghrelin system is a key component of emotionality responses. The precise mechanisms by which ghrelin controls mood remains unknown, but several brain regions important for regulation of emotional reactivity express the ghrelin receptor (Zigman et al., 2006; Alvarez-Crespo et al., 2012). Moreover, microinjections of ghrelin targeting the amygdala, hippocampus or dorsal raphe nucleus affect anxiety-like behaviors (Carlini et al., 2004).

Locus coeruleus (LC), however, is an unexplored substrate for ghrelin’s effects on behavior. One previous study mapping out brain nuclei accessible to ghrelin present in the cerebrospinal fluid (CSF) reported uptake of fluorescein-labeled ghrelin in the LC (Cabral et al., 2013). The LC is a small nucleus located deep in the brain stem, well-established as the major source of norepinephrine (NE) in the brain and critically involved in arousal, cognition and emotionality control (Poe et al., 2020). Yet, more recent work has also put forth the idea that the LC is involved in control of feeding behavior - LC activity is suppressed during feeding and in turn, LC stimulation suppresses food intake in mice (Sciolino et al., 2022). While this work puts LC on the map of brain substrates of food intake control, it remains to be shown which metabolic or endocrine signals feed into this brain region to allow its participation in food intake control. Given the role of LC in regulation of emotionality and feeding behavior control, along with the key role of ghrelin in both processes, and the fact that at least CSF ghrelin can access the nucleus, LC emerges as a potential direct brain target of ghrelin. To our knowledge, however, the ability of ghrelin to directly act in the rat LC and functional implications of this action have not yet been reported. In the present study, we investigated the LC as a potential novel target for ghrelin’s behavioral effects linked to appetite control, motivated behavior and emotional reactivity in male and female rats. We first sought to confirm the presence of GHSR in the LC of both sexes. Next, we determined the effects of LC GHSR pharmacological activation on food intake and motivated behavior for sucrose in male and female rats. Moreover, we determined whether ghrelin signaling in the LC modulates anxiety-like behavior, in both sexes. Finally, in order to determine whether LC GHSR activity is necessary for feeding and anxiety behavior control, we evaluated whether blockade of ghrelin receptors in the LC alters feeding, motivated, and anxiety behaviors. To increase the endogenous and physiological relevance of this question we utilized the newly identified endogenous GHSR antagonist - liver-expressed antimicrobial peptide 2 (LEAP2) (Ge et al., 2018; Schalla and Stengel, 2019). Endogenous LEAP2 levels in plasma are modulated by feeding status, such that they decrease during fasting and rise after feeding (Mani et al., 2019). It is a competitive antagonist and an inverse agonist of GHSR (Wang et al., 2019; M'Kadmi et al., 2019), making it a potent inhibitor of ghrelin signaling, yet still relatively unexplored.

## 2 Materials and methods

### 2.1 Animals

Female and male Sprague Dawley rats (8 weeks old upon arrival; Charles River Laboratories, Wilmington, MA and Charles River Laboratories, Italy) were individually housed on a 12-h light/dark cycle with *ad libitum* access to chow (PicoLab Rodent Diet 5053) and water. Drug injections and testing were performed during the light cycle. In the agonist experiments food was removed from the home cage at the time of drug injection, based on our previous findings that ghrelin’s anxiolytic effect may be abolished if rats are allowed to feed between ghrelin administration and anxiety testing (Alvarez-Crespo et al., 2012). In the antagonist experiments where overnight fasting was applied, the animals were food deprived at the onset of the dark cycle prior to drug injection and behavioral testing. After behavioral testing, rats were returned to their home cage with free access to chow, and food intake was measured 1 or 24 h after being returned. To test the effect of intra-LC ghrelin on the intake of palatable food, a high-fat high-sugar diet (HFHS, in-house made by mixing equal weight of lard and sugar) was offered together with chow and measured after 24 h. All procedures conformed to and received approval by Institutional Animal Care and Use Committee at the Pennsylvania State University and the Animal Welfare Committee of the University of Gothenburg, Sweden, Ethical permit # 137/15.

### 2.2 Stereotaxic surgery

Animals were anesthetized with an intraperitoneal injection of an anesthetic cocktail composed of ketamine (90 mg/kg), acepromazine (0.64 mg/kg), and xylazine (2.7 mg/kg). Analgesia (carprofen, 5 mg/kg) and local anesthesia (bupivacaine, 2.5 mg/kg) were administered subcutaneously prior to surgery. Guide cannula (26 gauge, 3 mm CC; P1 Technologies) targeting the LC were implanted (±1.3 mm from midline, 9.8 mm posterior to bregma, 5.2 mm ventral to skull, with injector aimed 7.2 mm ventral to skull; Paxinos and Watson, 2005) and affixed to the skull with bone screws and dental cement. Rats were given at least 1 week to recover from surgery before the start of behavioral testing.

### 2.3 *In situ* hybridization using RNAscope

*In situ* hybridization (ISH) using RNAscope^™^ Multiplex Fluorescent v2 kit (Advanced Cell Diagnostics) was utilized to determine presence of *Ghsr* (RNAscope™ Probe-Rn-Ghsr1a-C2, 431,991-C2) and *Th* (RNAscope™ Probe-Rn-Th, 314,651) mRNA in brain sections. To allow detection of colocalization, the two target probes were assigned to different probe channels and fluorophores. Fresh frozen brains were sectioned, and 12 μm thick coronal sections containing LC were collected and fixed in 4% formalin for 15 min at 4°C. Following two quick washes in PBS, brain slices were dehydrated in 50%, 70% and 2 × 100% ethanol (5 min each). Hydrogen peroxide was dropped on the slides and washed off after 10 min. Treatment with protease IV followed (30 min) and was washed away with PBS for a total time of 15 min. Target probes and negative control probes were applied directly on the sections to cover them completely and incubated at 40°C for 2 h in a hybridization oven. Following, slides were incubated with preamplifier and amplifier probes (AMP1, 40°C for 30 min; AMP2, 40°C for 15 min). Next, the HRP signal was developed (HRP-C1 and Opal dye 520; HRP-C2 and Opal dye 570). Finally, brain sections were incubated for 30 s with DAPI and followed by mounting medium for fluorescence (Vectashield). Slides were imaged with Olympus BX53 fluorescent microscope with cellSens imaging software.

### 2.4 RNA extraction and gene expression

Total RNA was extracted from the LC using the RNeasy Micro kit (Qiagen). cDNA was synthesized using the High-Capacity cDNA Reverse Transcription kit (Applied Biosystems). TaqMan gene expression kits and PCR reagents were used to quantify relative mRNA levels of GHSR (G*hsr*, Rn00821417_m1) relative to rat β-actin (*Actb*, Rn00667869_m1). Relative mRNA expression was calculated using the comparative ∆∆Ct method as previously described (Livak and Schmittgen, 2001).

### 2.5 Drugs

Ghrelin (Tocris) and rat LEAP2 (Phoenix Pharmaceuticals) were dissolved in artificial CSF (aCSF; Tocris), which was also used for the vehicle condition. Aliquots were stored at −20°C. Drugs were infused into the LC at a volume of 0.3 μL (flow rate 0.5 μL/min) and behavioral testing was conducted 20 min later, throughout the study. Ghrelin (1 μg) was administered unilaterally to *ad libitum-*fed rats at a dose that has previously been shown effective at increasing feeding behavior when injected into discrete brain sites (Schéle et al., 2016; López-Ferreras et al., 2017; Le May et al., 2019). LEAP2 (2.5 μg per hemisphere) was administered bilaterally to rats that were fasted overnight. The dose was derived from one of the few papers published with central injection of LEAP2, and a pre-print confirming the effectiveness on food intake based on a dose-response study (Islam et al., 2020; Tufvesson-Alm et al., 2023). We used low volumes for our parenchymal injection to the LC to prevent diffusion to the adjacent fourth ventricle, and spread to other brain areas that could be important for the observed behavioral effects. The antagonist (LEAP2) was injected bilaterally so that the activity from one hemisphere would not compensate for the loss of activity in the other. For all the experiments carried out in this study, rats were returned to their home cage with free access to chow after the behavioral testing. Acute food intake was measured after 1 h in the cage. In the case of all behavioral tests (operant conditioning, OF, ASR), drug injections were performed according to a cross-over balanced experimental design for each treatment separately. All conditions were separated by a minimum of 48 h wash out period and run in a counterbalanced manner (each rat received all conditions on separate testing days).

### 2.6 Operant conditioning

One distinctive aspect of reward is the motivation to self-administer or work for the reward (i.e., “wanting” the reward). The motivation to obtain a sucrose pellet (45 mg, Bio-serv) was assessed using the progressive ratio (PR) operant conditioning procedure (Hodos, 1961), a test measuring the number of lever presses that a rat is willing to execute to acquire a food reward. To prevent neophobia, rats were offered 3 sucrose pellets in their home cage the day before training was initiated. Training and testing were conducted in rat conditioning chambers (Med Associates) as described previously (Dickson et al., 2012). Rats were first trained on a 30-min fixed ratio schedules (FR1 followed by FR3 and FR5), where the cost of receiving one pellet equaled to 1, 3 and 5 presses on the active lever respectively. A minimum of 30 presses on the active lever per session was required for advancement to the next schedule. Finally, the rats were trained in 60-min PR conditioning sessions where the response requirement increased according to the following equation: response ratio = [5e(0.2 × infusion number)]—5 through the following series: 1, 2, 4, 9, 12, 15, 20, 25, 32, 40, 50, 62, 77, 95, 118, 145, 178, 219, 268, 328. The effect of drugs on food motivated behavior was tested with the PR schedule. Horizontal activity was measured with the use of infrared beams in the operant chambers. Food-seeking was measured by infrared beams that were responding to head entries into the pellet receptacle. The effect of ghrelin injection on food motivation was tested in *ad libitum* fed rats, while the effect of LEAP2 was tested in overnight fasted rats. This to ensure that the antagonist is applied when endogenous ghrelin levels are higher.

### 2.7 Open field

The open field test (OF) is based on the animal’s conflicting innate tendencies to avoid the open spaces and explore the novel environment. Administration of anxiolytics has been shown to increase time spent in the center of the open field, and decrease thigmotaxis (Walf and Frye, 2007). Here, rats were placed in the center of a brightly lit arena with dark walls (43.2 × 43.2 cm; Med Associates) and allowed to explore freely during 30 min. The animal’s position and movement were detected by a grid of photocells covering the arena, and the behavior was scored automatically using Med-Associates Activity Monitor software.

### 2.8 Acoustic startle response

The startle reflex is a primitive motor response to a sudden, intense stimulus, which is amplified in states of anxiety and diminished with anxiolytic drugs. In contrast to the open field test, the acoustic startle procedure can assess anxiety-like behavior without the influence of exploratory behavior and locomotor activity, components which may be affected by energy status and sex, and confound the interpretation of the results (Borchers et al., 2022). Testing was conducted in the SR-LAB startle response system (San Diego Instruments), a soundproof chamber with a cylindrical animal enclosure connected to a piezoelectric motion sensor that records the startle response. Rats were placed in the acrylic cylinder (9 cm in diameter) and habituated with a background white noise (50 dB) for 5 min. Following habituation, the SR Lab Software delivered acoustic stimuli bursts of 90, 95 or 105 dB (50 ms each) in a randomized pattern (10 times for each intensity) with inter stimulus intervals ranging between 20 and 40 s. Chambers were brightly lit during testing (500 lux illumination), as bright light acts as an unconditioned anxiogenic stimulus in rats (Walker and Davis, 1997). The peak amplitude response (in millivolts) to each sound stimulus (in dB) was averaged across the 10 repetitions and used as the dependent measure.

### 2.9 Tissue collection

Male and female rats were decapitated after light isoflurane anesthesia, and brains were rapidly removed, flash-frozen in isopentane, and stored at −80°C until processing. Half of the rats were fasted overnight prior to the euthanasia, while the other half had free access to chow, the groups were matched by body weight. Using a cryostat, coronal sections (12 μm thick) at the level of the LC were collected and slide-mounted for RNAscope *in situ hybridization*, micropunches of the LC were collected in test tubes for gene expression analysis, and sections were examined to ensure correct cannula placement. The rats that had cannulas placed outside of the LC in either hemisphere, were excluded from all behavioral analyses. For *Ghsr* expression studies, in addition to brains from rats used for behavioral testing, brains were also collected from a separate animal cohort matched for age, in order to increase the number of samples for reliable gene analysis.

### 2.10 Statistical analysis

All data are presented as mean ± SEM. Statistical significance was analyzed by two-factor repeated measures ANOVA with *post hoc* Holm–Sidak’s multiple comparison test when appropriate (GraphPad Prism 8 Software, Inc). To control for total locomotor activity in the OF, an analysis of covariance (ANCOVA) was performed using the *car* package for R (v. 4.3.1). *p*-values lower than 0.05 were considered statistically significant.

## 3 Results

### 3.1 Fluorescent *in situ* hybridization and qPCR show *Ghsr* expression in the rat LC

**FIGURE 1:**
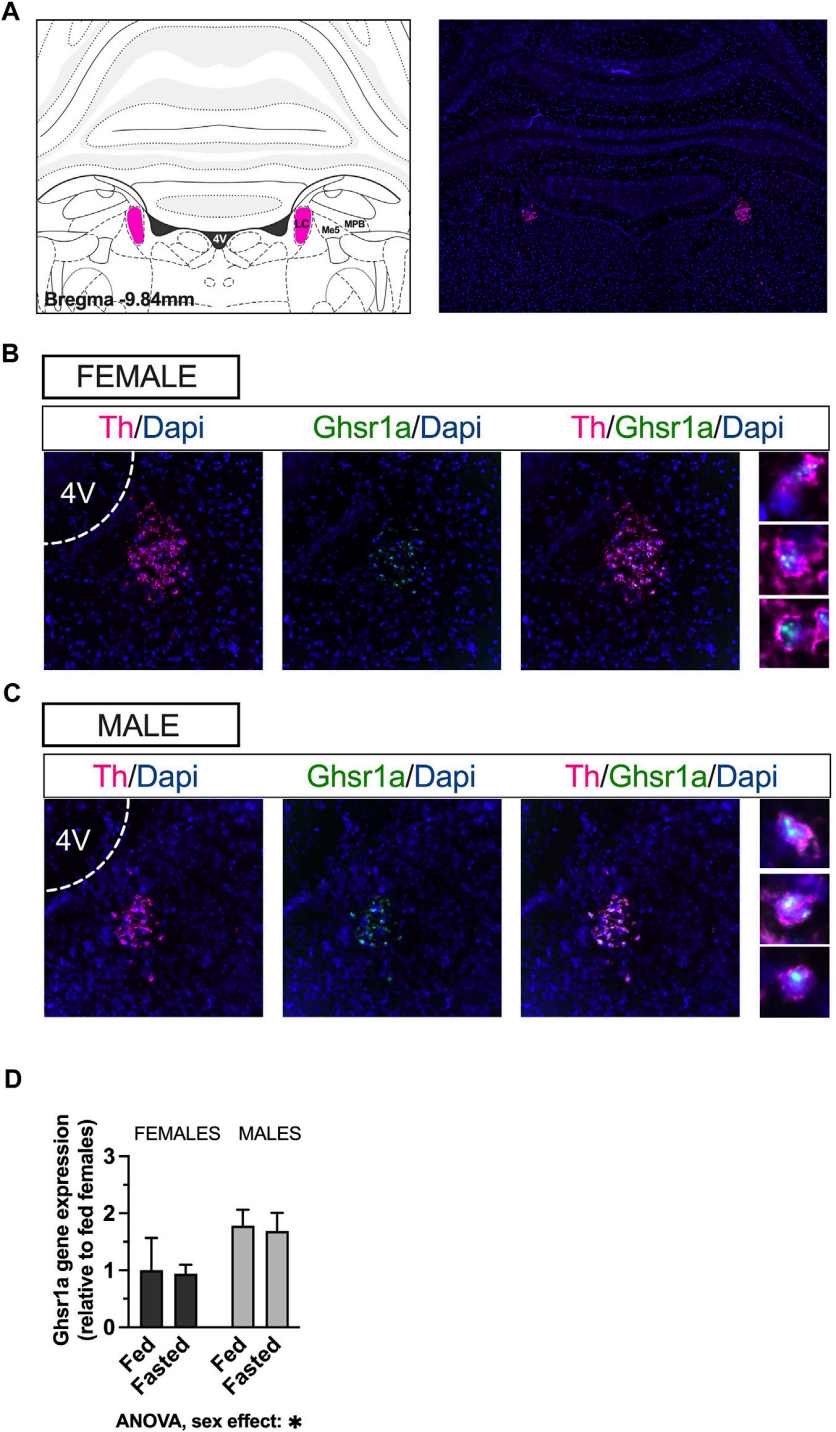
*Ghsr* are expressed in male and female LC, on noradrenergic neurons. RNAscope *in-situ* hybridization was used to determine expression of the *Ghsr* on *Th* neurons in LC. Representative image of *Th* mRNA (magenta) and cell nuclei (blue; DAPI) at the level of the LC in coronal brain sections of rats **(A)**. Co-localization indicates expression of GHSR1 (green) on *Th*-expressing cells (magenta) in the LC of female **(B)** and male **(C)** rats. qPCR performed on LC micropunches of *ad libitum* fed and fasted animals revealed that males expressed more *Ghsr* in the LC, but that the expression was unaffected by feeding status in both sexes **(D)**. Image corresponds to bregma −9.84 in Paxinos and Watson’s Rat Brain Atlas, fifth edition. LC, locus coeruleus; Me5, mesencephalic trigeminal nucleus; MPB, medial parabrachial nucleus; Th, tyrosine hydroxylase; Dapi, 4′,6-diamidino-2-phenylindole; Ghsr1a, growth hormone secretagogue receptor 1a; 4V, fourth ventricle.

To determine the presence of *Ghsr* in the LC and the presence of *Ghsr1a* transcript on LC noradrenergic neurons we performed RNAscope *in situ* hybridization (Figure 1A; representative image of a coronal brain section 9.8 mm posterior to bregma). Expression of *Ghsr* mRNA (green) was observed throughout the LC (Figures 1B, C). *Ghsr* was present on LC neurons that express tyrosine hydroxylase mRNA (*Th*) (magenta) in female (Figure 1B) and male (Figure 1C) rats, indicating presence of the ghrelin receptor on noradrenergic neurons. Moreover, we utilized real time quantitative PCR to measure the mRNA levels of *Ghsr* in *ad libitum* fed and fasted male and female rats. The gene expression analysis revealed that there was no effect of fasting on *Ghsr* expression, in either sex. However, the expression was sexually dimorphic, such that males had higher levels of ghrelin receptors in the LC [two-factor ANOVA: interaction *F*_(1, 79)_ = 0.001968, *p* = 0.9647, effect of fasting *F*_(1, 79)_ = 0.04919, *p* = 0.8250, effect of sex *F*_(1, 79)_ = 5.118, *p* = 0.0264; Figure 1D].

### 3.2 Pharmacological activation of LC GHSR stimulates food intake and food motivation in male and female rats

**FIGURE 2:**
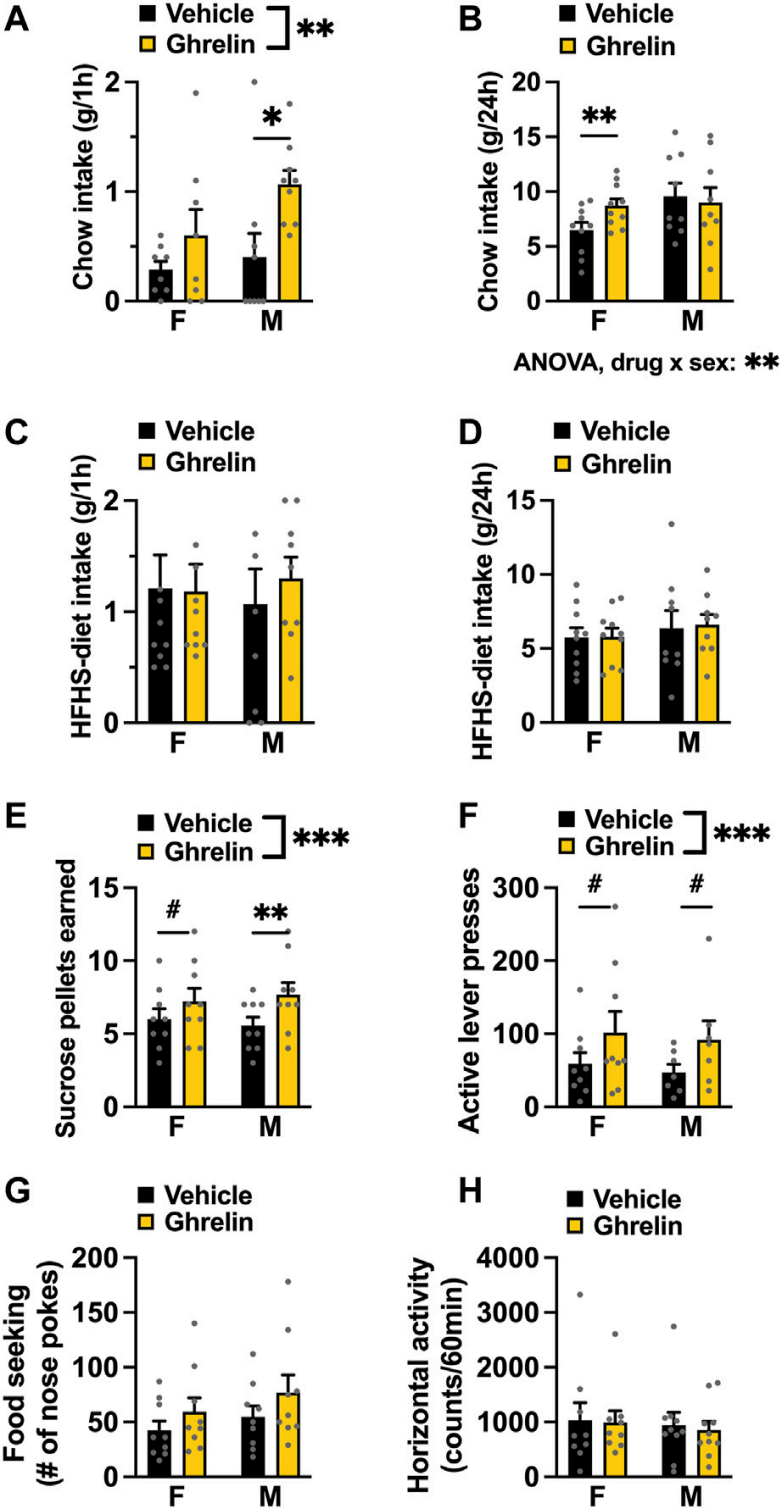
Ghrelin acts in the LC to increase chow intake and food reward in both female and male rats. Intra-LC administration of ghrelin (1 μg) ghrelin increased chow intake acutely in both males and females **(A)**, while at 24 h only females consumed more chow **(B)**. HFHS-diet intake remained unchanged 1 h **(C)** and 24 h **(D)** after ghrelin injection. In a progressive ratio operant schedule, ghrelin increased the amount of sucrose rewards earned **(E)** and the number of active lever presses to obtain the reward **(F)**, without changing food seeking **(G)** or locomotor activity **(H)** in either sex. F, females; M, males; HFHS diet, high-fat high-sugar diet. Data are expressed as mean ± SEM. #*p* < 0.1, *p < 0.05, **p < 0.01, ***p < 0.001, ****p < 0.0001.

Acute intra-LC ghrelin injection (1 μg) led to a significant increase in chow intake 1 h post injection [two-factor ANOVA for acute chow intake: interaction *F*_(1, 15)_ = 1.199, *p* = 0.2909, effect of drug *F*_(1, 15)_ = 9.161, *p* = 0.0085, effect of sex *F*_(1, 15)_ = 2.227, *p* = 0.1563, Figure 2A]. This hyperphagia persisted longer in females, which still had a greater chow intake at 24 h post ghrelin injection. Two-factor ANOVA revealed no significant effect of drug or sex, but a significant interaction between these two factors [two-factor ANOVA for 24 h food intake: interaction *F*_(1, 17)_ = 8.890, *p* = 0.0084, effect of drug *F*_(1, 17)_ = 3.228, *p* = 0.0902, effect of sex *F*_(1, 17)_ = 1.587, *p* = 0.2248; Figure 2B]. The effects of ghrelin on food intake were measured in a free choice paradigm, hence the animals were offered a palatable HFHS diet together with the chow. Here, ghrelin treatment did not affect HFHS-diet intake at any of the measured time points in either sex; [two-factor ANOVA for acute HFHS intake: interaction *F*_(1, 17)_ = 0.2277, *p* = 0.6393, effect of drug *F*_(1, 17)_ = 0.1358, *p* = 0.7171, effect of sex *F*_(1, 15)_ = 0.0019, *p* = 0.9654, Figure 2C], [two-factor ANOVA for 24 h HFHS intake: interaction *F*_(1, 17)_ = 0.0295, *p* = 0.8655, effect of drug *F*_(1, 17)_ = 0.0738, *p* = 0.7890, effect of sex *F*_(1, 17)_ = 0.5072, *p* = 0.4860, Figure 2D]. Applying ghrelin to the LC in *ad libitum* fed rats increased motivated behavior for a food reward–as evidenced by the higher amount of sucrose pellets earned [two-factor ANOVA: interaction *F*_(1, 16)_ = 1.174, *p* = 0.2946, effect of drug *F*_(1, 16)_ = 16.51, *p* = 0.0009, effect of sex *F*_(1, 16)_ = 0.000, *p* > 0.999; Figure 2E) due to the increased effort (active lever presses) rats were willing to expend for the reward [two-factor ANOVA: interaction *F*_(1, 14)_ = 0.003, *p* = 0.9549, effect of drug *F*_(1, 14)_ = 9.263, *p* = 0.0088, effect of sex *F*_(1, 14)_ = 0.141, *p* = 0.7125; Figure 2F]. The effect was specific to motivated behavior, as food seeking (Figure 2G) and locomotor activity (Figure 2H) were not affected, although there was a trend for effect of ghrelin on food seeking behavior [two-factor ANOVA: effect of drug *F*_(1, 16)_ = 3.214, *p* = 0.0919]. Both male and female rats responded to a similar extent as there was no significant drug‐sex interaction for any parameters measured during operant testing.

### 3.3 Blocking LC GHSR suppresses food intake and food motivation with a different latency in male and female rats

**FIGURE 3:**
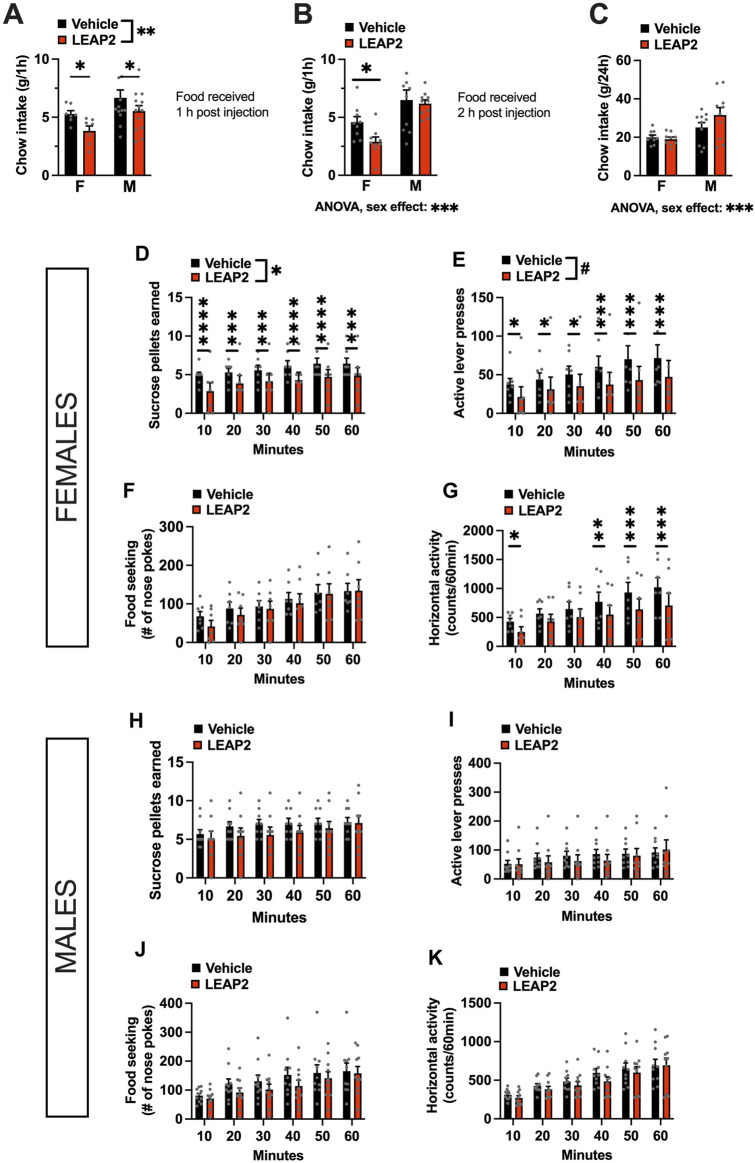
Acute pharmacological blockade of ghrelin signaling in the LC decreases food intake in both sexes, but food reward only in females. LEAP2 is anorexic in both sexes when chow is offered within 1 h post intra-LC injection **(A)**, but reduces chow intake in females only, when offered 2 hours post injection **(B)**. The anorexic effect of LEAP2 is absent in both sexes 24 h post intra-LC injection **(C)**. In females, intra-LC microinjection of LEAP2 (5 μg) decreased the number of sucrose pellets earned **(D)** and the number of lever presses for the rewards in a 60-min progressive ratio operant test **(E)**. Food seeking **(F)** was unchanged while locomotor activity was significantly reduced during the first 10 min and last 30 min of the test **(G)**. In contrast to females, acute pharmacological blockade of LC GHSR did not affect the number of sucrose pellets earned **(H)** or active lever presses **(I)** in males. Food seeking was also not significantly altered **(J)**. Locomotor activity in males was also not affected by the drug, **(K)**. LEAP2 = liver-expressed antimicrobial peptide 2. Data are expressed as mean ± SEM. #*p* < 0.1, *p < 0.05, **p < 0.01, ***p < 0.001, ****p < 0.0001.

Acute intra-LC LEAP2 injection (2.5 μg per hemisphere) in overnight fasted rats, significantly reduced feeding in both males and females when chow was offered to them 1 h after the antagonist administration and measured 1 h later [two-factor ANOVA: interaction *F*_(1, 18)_ = 0.1608, *p* = 0.6932 effect of drug *F*_(1, 18)_ = 11.27, *p* = 0.0035, effect of sex *F*_(1, 18)_ = 4.103, *p* = 0.0579; Figure 3A]. However, in a separate experiment when the pellets were returned to the animals 2 h after injection, *post hoc* analysis revealed that this hypophagia was only present in females [two-factor ANOVA: interaction *F*_(1, 17)_ = 1.786, *p* = 0.1991, effect of drug *F*_(1, 17)_ = 3.753, *p* = 0.0695, effect of sex *F*_(1, 17)_ = 17.13, *p* = 0.00079; Figure 3B]. At 24 h post injection, there were no effects of LEAP2 on chow intake [two-factor ANOVA: interaction *F*_(1, 17)_ = 1.473, *p* = 0.2414, effect of drug *F*_(1, 17)_ = 0.7907, *p* = 0.3863, effect of sex *F*_(1, 17)_ = 21.69, *p* = 0.0002; Figure 3C]. Intra-LC LEAP2 injection reduced motivated behavior in females as indicated by fewer sucrose rewards earned [two-factor ANOVA: interaction *F*_(5, 30)_ = 0.5021, *p* = 0.7722, effect of drug *F*_(1, 6)_ = 6.442, *p* = 0.0442, effect of time *F*_(5, 30)_ = 13.33, *p* < 0.0001; Figure 3D] throughout the entire test. There was a trend to reduction of lever presses [two-factor ANOVA: interaction *F*_(5, 30)_ = 1.195, *p* = 0.3354, effect of drug *F*_(1, 6)_ = 4.046, *p* = 0.0910, effect of time *F*_(5, 30)_ = 8.772, *p* < 0.0001; Figure 3E]. The interval between the number of presses required for each consecutive reward is amplified, creating a large variability for this parameter. However, since there was a clear reduction in the number of rewards that the females were willing to work for, we conclude that there was a drug effect on motivated behavior. Food seeking was not affected [two-factor ANOVA: interaction *F*_(5, 30)_ = 0.8984, *p* = 0.4949, effect of drug *F*_(1, 6)_ = 0.1929, *p* = 0.6759, effect of time *F*_(5, 30)_ = 21.51, *p* < 0.0001; Figure 3F]. Locomotor activity was also unaltered by the drug [two-factor ANOVA: interaction *F*_(5, 30)_ = 1.514, *p* = 0.2152, effect of drug *F*_(1, 6)_ = 3.184, *p* = 0.1246, effect of time *F*_(5, 30)_ = 18.39, *p* < 0.0001; Figure 3G]. In contrast to the consistent effect in females, behavior of LEAP2-injected male rats in the progressive ratio operant test was not significantly affected [two-factor ANOVA for rewards earned: interaction *F*_(5, 40)_ = 1.908, *p* = 0.1146, effect of drug *F*_(1, 8)_ = 0.9255, *p* = 0.3642, effect of time *F*_(5, 40)_ = 10.48, *p* < 0.0001; Figure 3H; two-factor ANOVA for lever presses: interaction *F*_(5, 40)_ = 1.228, *p* = 0.3141, effect of drug *F*_(1, 8)_ = 0.2433, *p* = 0.6351, effect of time *F*_(5, 40)_ = 8.664, *p* < 0.0001; Figure 3I; two-factor ANOVA for food seeking: interaction *F*_(5, 40)_ = 1.415, *p* = 0.2371, effect of drug *F*_(1, 8)_ = 1.246, *p* = 0.2932, effect of time *F*_(5, 40)_ = 11.89, *p* < 0.0001; Figure 3J]. Horizontal locomotor activity was not affected either [two-factor ANOVA: interaction *F*_(5, 40)_ = 1.114, *p* = 0.3539, effect of drug *F*_(1, 8)_ = 0.9553, *p* = 0.3539, effect of time *F*_(5, 40)_ = 27.87, *p* < 0.0001, Figure 3K].

### 3.4 Activation of LC GHSR alters anxiety-like behavior in a sex-specific manner

**FIGURE 4:**
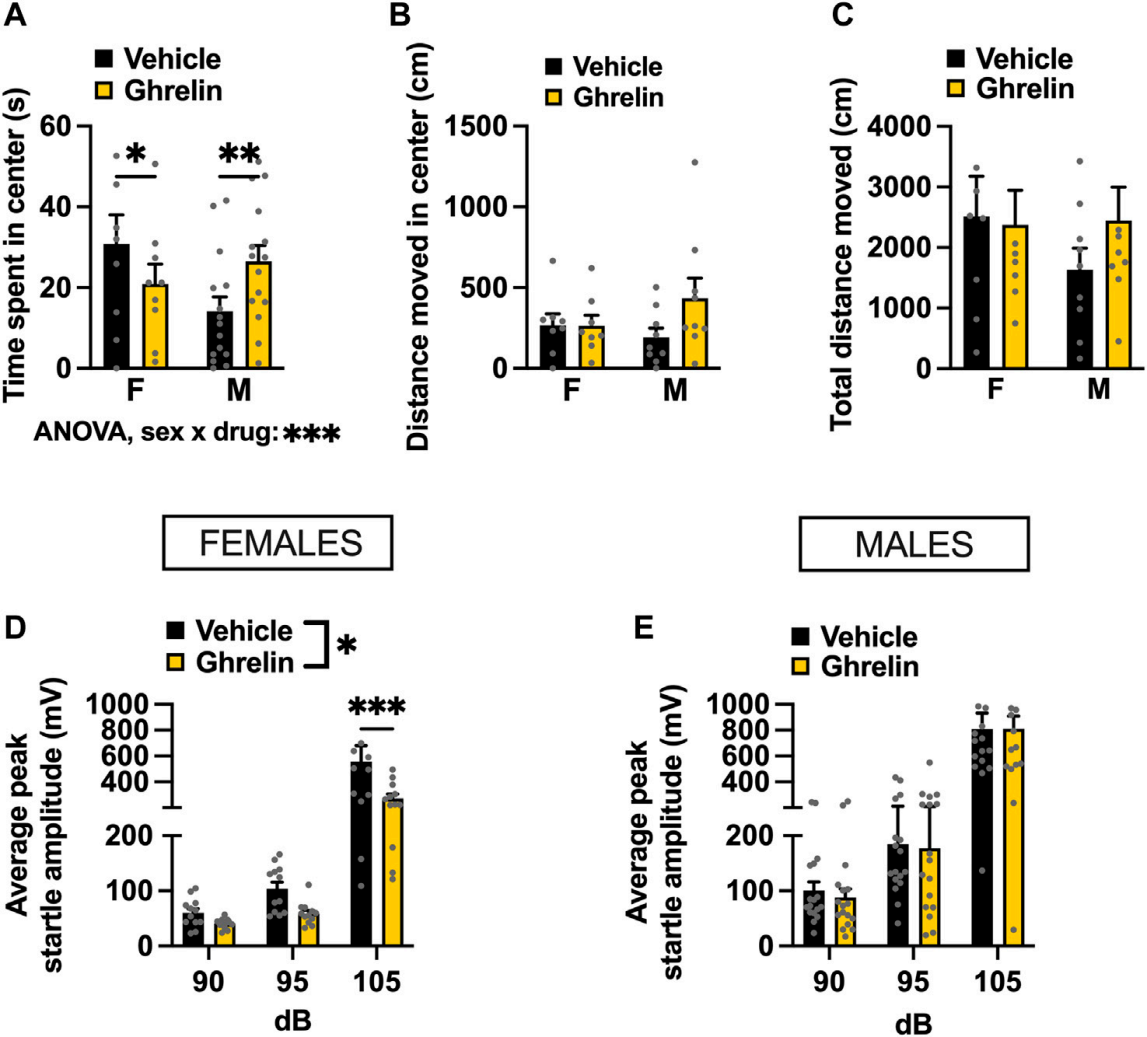
Ghrelin delivered to the LC alters anxiety-like behavior in a context-specific and sex-divergent manner. Intra-LC administration of ghrelin (1 μg) decreased the time females spent in the center, and conversely increased the time males spent in the center of the OF **(A)**. Distance moved in center **(B)** and total distance moved **(C)** remained unchanged, although a non-significant trend was detected in males. An anxiolytic effect of ghrelin was detected in the ASR test, where startle amplitude was significantly lower in ghrelin treated females **(D)** at the highest sound intensity. The treatment did not alter the startle response in males at any sound intensity **(E)**. LEAP2, liver-expressed antimicrobial peptide 2; F, females; M, males. Data are expressed as mean ± SEM. #*p* < 0.1, *p < 0.05, **p < 0.01, ***p < 0.001, ****p < 0.0001.

Initially, OF was used to determine the effects of intra-LC injection of ghrelin on anxiety-like behavior. In our paradigm, we withheld chow from the animals immediately before the drug injection, and did not return it until after behavioral testing, as our previous work indicates that offering food after ghrelin injections confounds the effect of this peptide on anxiety (Alvarez-Crespo et al., 2012). Interestingly, ghrelin treated females showed a potentially heightened anxiety-like behavior, based on less time spent in the center of the OF (Holm Šídák’s multiple comparisons test: *p* = 0.0259 Figure 4A). Males on the other hand, spent more time in the center of the OF after ghrelin injection, indicating a decreased anxiety-like behavior (Holm Šídák’s multiple comparisons test: *p* = 0.0018, Figure 4A). Two-factor ANOVA indicated a significant interaction between sex and drug effect [*F*_(1, 22)_ = 17.97, *p* = 0.0003], and no effect of drug [*F*_(1, 22)_ = 0.2143, *p* = 0.6479] or sex [*F*_(1, 22)_ = 0.7939, *p* = 0.3826] separately. While there was no significant change in locomotor activity in either sex, male rats appeared to move more, suggesting that the time spent in the center might be confounded by changes in general locomotor activity rather than emotional reactivity. Two-factor ANOVA indicated no significant effects for distance moved in center [two-factor ANOVA: interaction *F*_(1, 15)_ = 2.298, *p* = 0.1503, effect of drug *F*_(1, 15)_ = 2.166, *p* = 0.1618, effect of sex *F*_(1, 15)_ = 0.284, *p* = 0.6018; Figure 4B], nor total distance moved [two-factor ANOVA: interaction *F*_(1, 15)_ = 0.6912, *p* = 0.4188, effect of drug *F*_(1, 15)_ = 0.3461, *p* = 0.5651, effect of sex *F*_(1, 15)_ = 0.6594, *p* = 0.4295; Figure 4C]. To explore the influence of locomotion on anxiety behavior in OF statistically, we performed an ANCOVA and determined the relationship between the total distance travelled in the arena and time in the center of the open field. In males, the covariate, total distance did influence time spent in the center of the open field [*F*_(1,21)_ = 12.863, *p* < 0.001]. There was no significant effect of the treatment on the time spent in the center after controlling for the effect of total locomotor activity [*F*_(1,21)_ = 0.010, *p* = 0.9204]. In females on the other hand, neither total locomotor activity nor treatment had a significant relationship to the time spent in the center with *F*_(1,13)_ = 0.997, *p* = 0.3362 and *F*_(1,13)_ = 0.897, *p* = 0.3606, respectively. Overall, the ANCOVA results indicated that the effect of treatment was not significant after controlling for total activity in both sexes. In order to further separate potential influence of locomotion from assessment of anxiety‐like behavior we performed the ASR test. The ASR clearly revealed an anxiolytic effect of ghrelin in females, and as expected an effect of the increasing sound intensity, which plays the role of an anxiogenic stimulus in the ASR test [two-factor ANOVA: interaction *F*_(2, 22)_ = 5.092, *p* = 0.0152, effect of drug *F*_(1, 11)_ = 8.003, *p* = 0.0164, effect of sound *F*_(2, 22)_ = 26.270, *p* < 0.0001; Figure 4D]. In males, there was no drug effect at any sound intensity [two-factor ANOVA: interaction *F*_(2, 32)_ = 0.0119, *p* = 0.9881, effect of drug *F*_(1, 16)_ = 0.0201, *p* = 0.8890, effect of sound *F*_(2, 32)_ = 60.03 *p* < 0.0001; Figure 4E].

### 3.5 Intra-LC administration of LEAP2 exerts an anxiogenic effect

**FIGURE 5:**
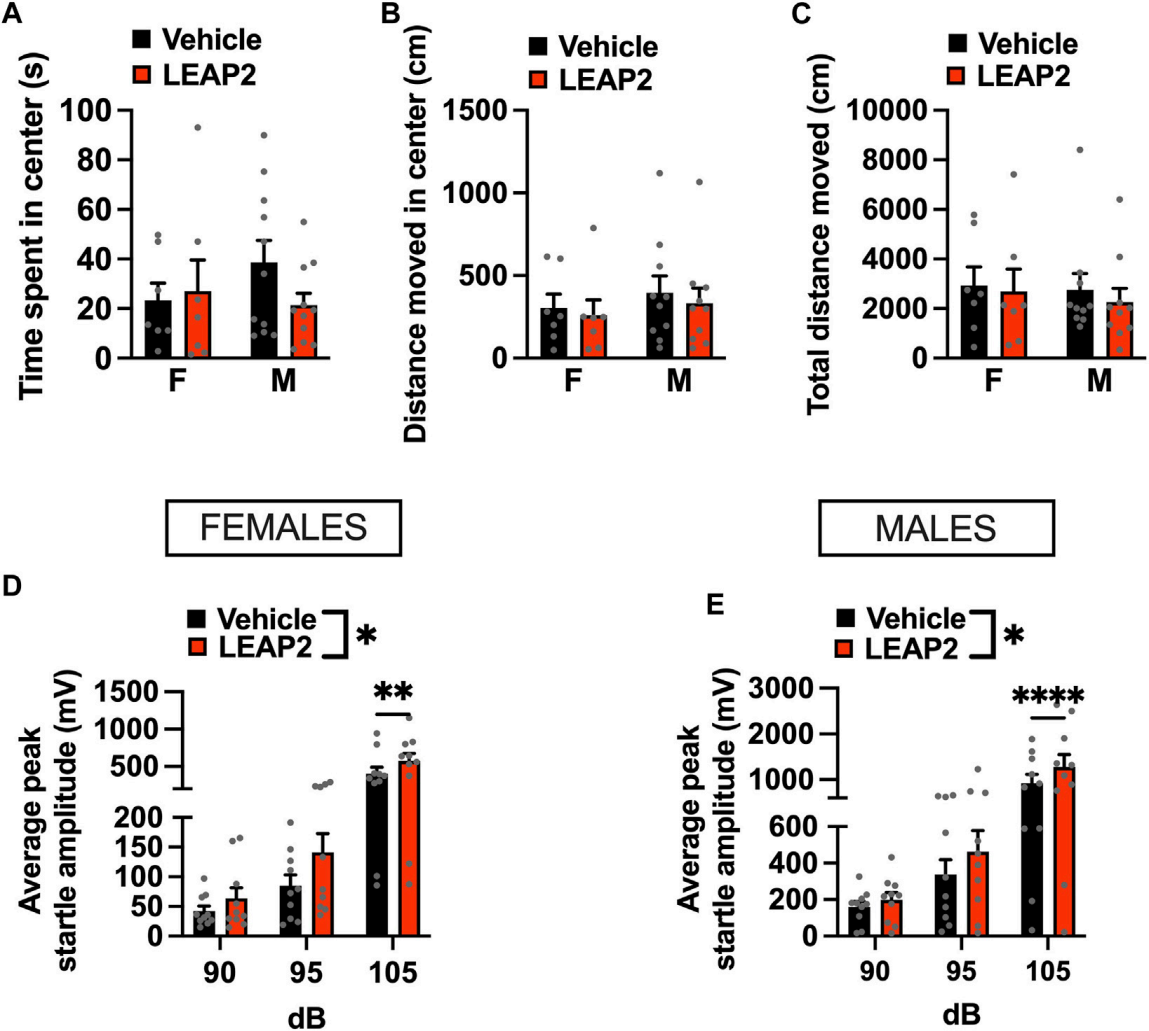
Acute pharmacological blockade of GHSR increases anxiety-like behavior in male and female rats. Intra-LC microinjection of LEAP2 (5 μg) did not change time spent in center **(A)**, locomotor activity in center **(B)**, or total distance moved **(C)** in the OF test. However, an anxiogenic effect of ghrelin receptor blockade in LC was detected in the ASR test, where startle amplitude, was significantly increased in LEAP2 treated females **(D)** and males **(E)**. LEAP2 = liver-expressed antimicrobial peptide 2; F, females; M, males. Data are expressed as mean ± SEM. #*p* < 0.1, *p < 0.05, **p < 0.01, ***p < 0.001, ****p < 0.0001.

To determine if ghrelin signaling in the LC is necessary for anxiety-like behavior, we exposed fasted rats to the OF and ASR tests after LEAP2 administration. LEAP2 did not alter time spent in the center [interaction *F*_(1, 16)_ = 2.285, *p* = 0.1502, effect of drug *F*_(1, 16)_ = 0.939, *p* = 0.3468, effect of sex *F*_(1, 16)_ = 0.241, *p* = 0.6301; Figure 5A], distance moved in center [interaction *F*_(1, 15)_ = 0.0407, *p* = 0.8428, effect of drug *F*_(1, 15)_ = 1.637, *p* = 0.2202, effect of sex *F*_(1, 15)_ = 0.395, *p* = 0.5388; Figure 5B] nor total distance moved in the OF [interaction *F*_(1, 15)_ = 0.124, *p* = 0.7294, effect of drug *F*_(1, 15)_ = 0.918, *p* = 0.3531, effect of sex *F*_(1, 15)_ = 0.106, *p* = 0.7484; Figure 5C].

As fasting and manipulating ghrelin signaling may influence locomotor activity, we performed an ANCOVA for time spent in the center with total distance travelled in the open field as a covariate. The covariate, total locomotion, was significantly related to the time males spent in the center of the OF (*F*_(1,17)_ = 14.139, *p* < 0.001). After controlling for locomotion, there was no significant effect of LEAP2 on the time spent in the center of the OF (*F*_(1,17)_ = 0.024, *p* = 0.8783). Similar to males, an effect of total locomotion on time spent in the open areas of the OF (*F*_(1,11)_ = 42.03, *p* < 0.0001) was found in females. LEAP2 treatment had no effect on the time spent in the center of the OF after controlling for locomotion (*F*_(1,11)_ = 0.849, *p* = 0.3764). Therefore, LEAP2 did not have an effect on anxiety-like behavior in the OF after adjusting for locomotion, in either sex.

In contrast, LEAP2 was clearly anxiogenic in both females (Figure 5D) and males (Figure 5E) when tested in the ASR. A two-factor repeated measures ANOVA for the female data indicated an effect of sound intensity [*F*_(2, 18)_ = 26.23, *p* < 0.0001], drug [*F*_(1, 9)_ = 10.50, *p* < 0.0101] and a strong trend for an interaction between the two factors [*F*_(2, 18)_ = 3.371, *p* < 0.0571]. In males, two-factor repeated measures ANOVA revealed an effect of sound intensity [*F*_(2, 18)_ = 8.914, *p* < 0.002], drug [*F*_(1, 9)_ = 8.882, *p* < 0.0154] and a significant interaction [*F*_(2, 18)_ = 8.914, *p* < 0.002].

## 4 Discussion

In the present study, we set out to determine whether ghrelin action in the LC is necessary and sufficient for control of ingestive, motivated, and emotionality behaviors. To achieve this, we investigated the behavioral outputs of pharmacological activation and inhibition of ghrelin receptors in the LC. We found that GHSR are present on noradrenergic cells in the LC of male and female rats, with higher levels in males. We show that intra-LC-injected ghrelin acutely increases food intake and also motivated behavior for sucrose. In contrast, blockade of GHSR, by intra-LC LEAP2 microinjections performed in fasted rats in order to ensure high levels of endogenously produced circulating ghrelin, resulted in decreased feeding and reduced food reward in females, while males only presented with transiently suppressed food intake. Activation of LC ghrelin receptors was anxiolytic in female rats, as indicated by decreased startle responses in the ASR test. Conversely, blockade of LC ghrelin receptors was anxiogenic in females. In males, activation of LC ghrelin receptors had no effect on behavior in the ASR test, however blockade was anxiogenic. These data indicate that the LC is a novel brain area key to ghrelin’s effects on ingestive, motivated, and emotionality behavior, with some sex divergence in these effects.

Ghrelin has a well-established orexigenic effect, consistently demonstrated in the literature by peripheral injections and direct application to a variety of GHSR expressing brain areas (Faulconbridge et al., 2008; Egecioglu et al., 2010; Skibicka and Dickson, 2011; Mason et al., 2014; Wald et al., 2023). Here, we show that exogenous ghrelin increases chow intake and food motivated behavior at the level of LC as well. Acutely, we find no sex differences in chow intake measured over 1 h, nor motivated behavior for a sucrose pellet. Surprisingly, at 24 h post injection our analysis indicates an interaction between sex and treatment for the orexigenic effect, as females still had a significantly higher chow intake when measured the day after intra-LC injection. According to studies done in male rodents, most of ghrelin’s hyperphagic effect takes place within 3 h, and central injection of ghrelin is reported to have no effect on feeding at 24 h post drug application in males (Faulconbridge et al., 2003; Skibicka et al., 2012). The more persistent effect on food intake that we describe in the current study is unusual but consistent with data from ghrelin injections into the LH, which exert a female-specific hyperphagia 24 h after administration (López-Ferreras et al., 2017). In contrast to the current results, we previously found the expression of *Ghsr* in the LH is higher in females (Börchers et al., 2022b). Notably, while intra-LC ghrelin increased the motivation to work for a sucrose pellet, it did not induce HFHS-intake in a free choice paradigm. Previous reports have demonstrated that central injection of ghrelin mainly induces a preference for chow over lard and sucrose (Schéle et al., 2016), and increases motivated behavior similarly for regular chow and palatable treats (Bake et al., 2019). Results of ghrelin receptor blockade at the level of LC unveiled further differences between sexes. While LEAP2 was effective in reducing fasting-induced intake and fasting-potentiated food reward in females at all tested time points, it had a transient effect on ingestive behavior in males. Moreover, motivated behavior was not significantly affected by LEAP2 in males, suggesting that the ghrelin system is necessary in females but not males in LC for control of food motivated behavior. It is likely that in males, fasting-induced activity at other ghrelin receptor-expressing brain sites is sufficient to compensate for the blocked ghrelinergic signaling from the LC (Zigman et al., 2006; Skibicka et al., 2011; Mason et al., 2014; Uriarte et al., 2019), or that there may be sex differences in one or several of the molecular components of the ghrelin-axis at the level of LC. Previous work from our group, and others, suggests that the ghrelin axis is sexually dimorphic in rats–females have much higher circulating ghrelin levels at baseline and after overnight fasting, less hepatic LEAP2, and higher ghrelin receptor expression in emotionality-influencing brain areas (Borchers et al., 2022a; Smith et al., 2022). Here, we demonstrate sex differences in *Ghsr* expression in the LC, with higher levels expressed in males, providing a potential explanation and molecular basis to the observed behavioral differences in response to LEAP2. The ghrelin receptor has a high constitutive activity, allowing it to maintain energy homeostasis without ghrelin binding (Fernandez et al., 2018). LEAP2 is a competitive antagonist and an inverse agonist of the GHSR, and sexually dimorphic expression of the receptor in the LC is very likely to influence pharmacological response at this site (M'Kadmi et al., 2019). However, a future dose-response study is needed to confirm this hypothesis, along with complementary analysis of the other components of the ghrelin axis.

Consistently with the sex divergent effects on feeding, we show a female-specific anxiolysis after intra-LC administration of ghrelin as indicated by the ASR test. This supports the hypothesis that female rats are more sensitive to the anxiolytic effects of ghrelin signaling, as a survival mechanism during periods of negative energy balance (Borchers et al., 2022a). The ability of the brain to integrate internal physiological drives, such as hunger, with external stimuli is essential for survival. LC is important for regulation of arousal, and is activated by unexpected sensory events to direct attention to potentially threatening stimuli in the environment (Aston-Jones and Cohen, 2005). Psychiatric disorders linked to stress and hyperarousal, namely, post-traumatic stress disorder (PTSD), anxiety and major depression are more prevalent in women than men (McLean et al., 2011; Olff, 2017; Li et al., 2022). Interestingly, there are well-established sex differences in the anatomy and physiology of the LC, biasing female rodents towards increased arousal and dysregulation by stress (Bangasser et al., 2016). Hunger state has been shown to affect LC response to unexpected stimuli, as a light flash evoked a greater activation of LC-NE neurons in fasted mice compared to satiated mice (Sciolino et al., 2022). Additionally, the LC has been implicated in the control of fear-induced suppression of eating through co-release of NE and glutamate in response to threatening stimuli (Yang et al., 2021). It is therefore conceivable that ghrelin, the circulating hunger hormone, modulates LC activity to address hunger rather than fear when appropriate, and that sensitivity in this system is higher in females.

Some studies indicate that plasma ghrelin may not access many of the brain regions expressing GHSR (Cabral et al., 2014; Perello et al., 2019). However, the ability of endogenous ghrelin to reach its receptors at the LC level is supported by its location next to the fourth ventricle, along with the finding that systemic ghrelin administration increases CSF ghrelin (Uriarte et al., 2019), and one previous report showing the uptake of CSF ghrelin in the LC (Cabral et al., 2013). Our *in-situ* hybridization and qPCR results confirm that *Ghsr* mRNA is present in the LC. The *Ghsr-*expressing cells were distributed within the entire nucleus and lacked any evident cluster organization in both sexes. Importantly, we show that *Ghsr* is co-expressed with *Th*, placing the receptor on norepinephrine-producing cells in the LC. The LC is the major source of noradrenergic ascending fibers to the forebrain, but investigations into the role of brain NE circuits in feeding have not conveyed uniform results. For instance, catecholamine projections from the (NTS) to the (Arc) were shown to stimulate feeding, while activating projections to the parabrachial nucleus (PBN) suppress feeding (Roman et al., 2016; Aklan et al., 2020). Moreover, lesions of the ventral noradrenergic bundle result in hyperphagia, while the effect on ingestive behavior following the interruption of projections of the dorsal noradrenergic bundle is less clear (Ahlskog and Hoebel, 1973; Sahakian et al., 1983). One previous study indirectly supports a potential functional interaction of ghrelin with catecholaminergic neurons: Chuang *et al.* found that ghrelin signaling in *Th*-expressing neurons was sufficient to mediate ghrelin’s orexigenic, antidepressant-like, and stress-induced food-reward behavior (Chuang et al., 2011). The authors discuss the probable involvement of GHSR in VTA for the effects observed in their *Th*-*cre* mouse model, however the exact neural site responsible for the phenotype was not investigated in this study. Put together with our results, it is intriguing to speculate that the direct action of ghrelin on GHSR-TH neurons in the LC could be mediating this stress-associated feeding behavior. However, Sciolino *et al.* recently demonstrated that activation of noradrenergic neurons in the LC attenuates food intake, and identified a circuit from the LC to LH that modulates feeding and anxiety-like behavior (Sciolino et al., 2022). It is possible that ghrelin receptors and ghrelin provide an upstream signal suppressing noradrenergic neurons and the LC-LH circuits. Future studies using viral tracing could indicate whether GHSR can be found on these specific neurons. However, given the broad and relatively uniform distribution of *Ghsr* expression throughout the LC it is likely that ghrelin indeed controls this circuit. The central role of the LC in integrating physiological drives and external stimuli, underscores its position as a pivotal brain structure in orchestrating complex behaviors necessary for survival. Here, we identify the LC as a novel target site for ghrelin’s influence on the brain. Our investigation sheds light on how the stomach, through the actions of ghrelin, can exert direct effect on behaviors that the LC governs, providing an important link between energy homeostasis and emotional states. Ghrelin-signaling pathways also affect motivation and ingestion of artificial rewards (Jerlhag et al., 2009; Jerlhag et al., 2010), and the LC plays an important role in substance abuse disorder (Van Bockstaele et al., 2010), making it compelling to speculate that GHSR signaling in the LC could also mediate reward for substances of abuse. Importantly, our study highlights sex-specific responses to ghrelin signaling in the LC, offering a framework for further investigation into the underlying neural circuitry and the molecular pathways driving these divergent effects. Given the involvement of both ghrelin and LC-NE in stress-related psychiatric disorders, along with the presence of sex-specific differences in these systems, it is crucial to gain a comprehensive understanding of their interaction. This knowledge could potentially contribute to the development of sex-tailored therapies for eating- and anxiety disorders.

## Data Availability

The raw data supporting the conclusion of this article will be made available by the authors, without undue reservation.

## References

[B1] Abizaid A. Liu Z. W. Andrews Z. B. Shanabrough M. Borok E. Elsworth J. D. (2006). Ghrelin modulates the activity and synaptic input organization of midbrain dopamine neurons while promoting appetite. J. Clin. Invest 116, 3229–3239. 10.1172/JCI29867 17060947 PMC1618869

[B2] Ahlskog J. E. Hoebel B. G. (1973). Overeating and obesity from damage to a noradrenergic system in the brain. Science 182, 166–169. 10.1126/science.182.4108.166 4517136

[B3] Aklan I. Sayar Atasoy N. Yavuz Y. Ates T. Coban I. Koksalar F. (2020). NTS catecholamine neurons mediate hypoglycemic hunger via medial hypothalamic feeding pathways. Cell Metab. 31, 313–326. 10.1016/j.cmet.2019.11.016 31839488 PMC9017597

[B4] Alvarez-Crespo M. Skibicka K. P. Farkas I. Molnár C. S. Egecioglu E. Hrabovszky E. (2012). The amygdala as a neurobiological target for ghrelin in rats: neuroanatomical, electrophysiological and behavioral evidence. PLoS One 7, e46321. 10.1371/journal.pone.0046321 23071554 PMC3468604

[B5] Andrews Z. B. (2011). Central mechanisms involved in the orexigenic actions of ghrelin. Peptides 32, 2248–2255. 10.1016/j.peptides.2011.05.014 21619904

[B6] Asakawa A. Inui A. Kaga T. Yuzuriha H. Nagata T. Fujimiya M. (2001). A role of ghrelin in neuroendocrine and behavioral responses to stress in mice. Neuroendocrinology 74, 143–147. 10.1159/000054680 11528215

[B7] Aston-Jones G. Cohen J. D. (2005). An integrative theory of locus coeruleus-norepinephrine function: adaptive gain and optimal performance. Annu. Rev. Neurosci. 28, 403–450. 10.1146/annurev.neuro.28.061604.135709 16022602

[B8] Bake T. Edvardsson C. E. Cummings C. J. Dickson S. L. (2019). Ghrelin's effects on food motivation in rats are not limited to palatable foods. J. Neuroendocrinol. 31, e12665. 10.1111/jne.12665 30525248 PMC6767751

[B9] Bangasser D. A. Wiersielis K. R. Khantsis S. (2016). Sex differences in the locus coeruleus-norepinephrine system and its regulation by stress. Brain Res. 1641, 177–188. 10.1016/j.brainres.2015.11.021 26607253 PMC4875880

[B10] Barrile F. Cassano D. Fernandez G. De Francesco P. N. Reynaldo M. Cantel S. (2023). Ghrelin’s orexigenic action in the lateral hypothalamic area involves indirect recruitment of orexin neurons and arcuate nucleus activation. Psychoneuroendocrinology 156, 106333. 10.1016/j.psyneuen.2023.106333 37454647 PMC10530520

[B11] Borchers S. Krieger J. P. Asker M. Maric I. Skibicka K. P. (2022). Commonly-used rodent tests of anxiety-like behavior lack predictive validity for human sex differences. Psychoneuroendocrinology 141, 105733. 10.1016/j.psyneuen.2022.105733 35367714

[B12] Borchers S. Krieger J. P. Maric I. Carl J. Abraham M. Longo F. (2022a). From an empty stomach to anxiolysis: molecular and behavioral assessment of sex differences in the ghrelin Axis of rats. Front. Endocrinol. (Lausanne) 13, 901669. 10.3389/fendo.2022.901669 35784535 PMC9243305

[B13] Börchers S. Krieger J. P. Maric I. Carl J. Abraham M. Longo F. (2022b). From an empty stomach to anxiolysis: molecular and behavioral assessment of sex differences in the ghrelin Axis of rats. Front. Endocrinol. (Lausanne) 13, 901669. 10.3389/fendo.2022.901669 35784535 PMC9243305

[B14] Cabral A. Fernandez G. Perello M. (2013). Analysis of brain nuclei accessible to ghrelin present in the cerebrospinal fluid. Neuroscience 253, 406–415. 10.1016/j.neuroscience.2013.09.008 24042041 PMC3850106

[B15] Cabral A. Valdivia S. Fernandez G. Reynaldo M. Perello M. (2014). Divergent neuronal circuitries underlying acute orexigenic effects of peripheral or central ghrelin: critical role of brain accessibility. J. Neuroendocrinol. 26, 542–554. 10.1111/jne.12168 24888783 PMC4108543

[B16] Carlini V. P. Monzón M. E. Varas M. M. Cragnolini A. B. Schiöth H. B. Scimonelli T. N. (2002). Ghrelin increases anxiety-like behavior and memory retention in rats. Biochem. biophysical Res. Commun. 299, 739–743. 10.1016/s0006-291x(02)02740-7 12470640

[B17] Carlini V. P. Varas M. M. Cragnolini A. B. Schiöth H. B. Scimonelli T. N. de Barioglio S. R. (2004). Differential role of the hippocampus, amygdala, and dorsal raphe nucleus in regulating feeding, memory, and anxiety-like behavioral responses to ghrelin. Biochem. biophysical Res. Commun. 313, 635–641. 10.1016/j.bbrc.2003.11.150 14697239

[B18] Chuang J.-C. Perello M. Sakata I. Osborne-Lawrence S. Savitt J. M. Lutter M. (2011). Ghrelin mediates stress-induced food-reward behavior in mice. J. Clin. Investigation 121, 2684–2692. 10.1172/JCI57660 PMC3223843 21701068

[B19] Chuang J. C. Zigman J. M. (2010). Ghrelin's roles in stress, mood, and anxiety regulation. Int. J. Pept. 2010, 460549. 10.1155/2010/460549 20721341 PMC2915752

[B20] Cummings D. E. Purnell J. Q. Frayo R. S. Schmidova K. Wisse B. E. Weigle D. S. (2001). A preprandial rise in plasma ghrelin levels suggests a role in meal initiation in humans. Diabetes 50, 1714–1719. 10.2337/diabetes.50.8.1714 11473029

[B21] Dickson S. L. Shirazi R. H. Hansson C. Bergquist F. Nissbrandt H. Skibicka K. P. (2012). The glucagon-like peptide 1 (GLP-1) analogue, exendin-4, decreases the rewarding value of food: a new role for mesolimbic GLP-1 receptors. J. Neurosci. 32, 4812–4820. 10.1523/JNEUROSCI.6326-11.2012 22492036 PMC6620919

[B22] Egecioglu E. Jerlhag E. Salomé N. Skibicka K. P. Haage D. Bohlooly-Y M. (2010). Ghrelin increases intake of rewarding food in rodents. Addict. Biol. 15, 304–311. 10.1111/j.1369-1600.2010.00216.x 20477752 PMC2901520

[B23] Faulconbridge L. F. Cummings D. E. Kaplan J. M. Grill H. J. (2003). Hyperphagic effects of brainstem ghrelin administration. Diabetes 52, 2260–2265. 10.2337/diabetes.52.9.2260 12941764

[B24] Faulconbridge L. F. Grill H. J. Kaplan J. M. Daniels D. (2008). Caudal brainstem delivery of ghrelin induces fos expression in the nucleus of the solitary tract, but not in the arcuate or paraventricular nuclei of the hypothalamus. Brain Res. 1218, 151–157. 10.1016/j.brainres.2008.04.068 18514175 PMC2528066

[B25] Fernandez G. Cabral A. Andreoli M. F. Labarthe A. M'Kadmi C. Ramos J. G. (2018). Evidence supporting a role for constitutive ghrelin receptor signaling in fasting-induced hyperphagia in male mice. Endocrinology 159, 1021–1034. 10.1210/en.2017-03101 29300858

[B26] Ge X. Yang H. Bednarek M. A. Galon-Tilleman H. Chen P. Chen M. (2018). LEAP2 is an endogenous antagonist of the ghrelin receptor. Cell metab. 27, 461–469. 10.1016/j.cmet.2017.10.016 29233536

[B27] Hodos W. (1961). Progressive ratio as a measure of reward strength. Science 134, 943–944. 10.1126/science.134.3483.943 13714876

[B28] Hyland L. Park S. B. Abdelaziz Y. Abizaid A. (2020). Ghrelin infused into the dorsomedial hypothalamus of male mice increases food intake and adiposity. Physiol. Behav. 220, 112882. 10.1016/j.physbeh.2020.112882 32205145

[B29] Islam M. N. Mita Y. Maruyama K. Tanida R. Zhang W. Sakoda H. (2020). Liver-expressed antimicrobial peptide 2 antagonizes the effect of ghrelin in rodents. J. Endocrinol. 244, 13–23. 10.1530/JOE-19-0102 31539874 PMC6839046

[B30] Jerlhag E. Egecioglu E. Dickson S. L. Engel J. A. (2010). Ghrelin receptor antagonism attenuates cocaine- and amphetamine-induced locomotor stimulation, accumbal dopamine release, and conditioned place preference. Psychopharmacol. Berl. 211, 415–422. 10.1007/s00213-010-1907-7 PMC2908453 20559820

[B31] Jerlhag E. Egecioglu E. Landgren S. Salomé N. Heilig M. Moechars D. (2009). Requirement of central ghrelin signaling for alcohol reward. Proc. Natl. Acad. Sci. U. S. A. 106, 11318–11323. 10.1073/pnas.0812809106 19564604 PMC2703665

[B32] Kojima M. Hosoda H. Date Y. Nakazato M. Matsuo H. Kangawa K. (1999). Ghrelin is a growth-hormone-releasing acylated peptide from stomach. Nature 402, 656–660. 10.1038/45230 10604470

[B33] Kristenssson E. Sundqvist M. Astin M. Kjerling M. Mattsson H. Dornonville de la Cour C. (2006). Acute psychological stress raises plasma ghrelin in the rat. Regul. Pept. 134, 114–117. 10.1016/j.regpep.2006.02.003 16540188

[B34] Le May M. V. Hume C. Sabatier N. Schéle E. Bake T. Bergström U. (2019). Activation of the rat hypothalamic supramammillary nucleus by food anticipation, food restriction or ghrelin administration. J. Neuroendocrinol. 31, e12676. 10.1111/jne.12676 30580497

[B35] Li S. Xu Y. Zheng L. Pang H. Zhang Q. Lou L. (2022). Sex difference in global burden of major depressive disorder: findings from the global burden of disease study 2019. Front. Psychiatry 13, 789305. 10.3389/fpsyt.2022.789305 35264985 PMC8898927

[B36] Livak K. J. Schmittgen T. D. (2001). Analysis of relative gene expression data using real-time quantitative PCR and the 2(-Delta Delta C(T)) Method. methods 25, 402–408. 10.1006/meth.2001.1262 11846609

[B37] López-Ferreras L. Richard J. E. Anderberg R. H. Nilsson F. H. Olandersson K. Kanoski S. E. (2017). Ghrelin's control of food reward and body weight in the lateral hypothalamic area is sexually dimorphic. Physiology Behav. 176, 40–49. 10.1016/j.physbeh.2017.02.011 PMC5433916 28213203

[B38] Lutter M. Sakata I. Osborne-Lawrence S. Rovinsky S. A. Anderson J. G. Jung S. (2008). The orexigenic hormone ghrelin defends against depressive symptoms of chronic stress. Nat. Neurosci. 11, 752–753. 10.1038/nn.2139 18552842 PMC2765052

[B39] Mani B. K. Puzziferri N. He Z. Rodriguez J. A. Osborne-Lawrence S. Metzger N. P. (2019). LEAP2 changes with body mass and food intake in humans and mice. J. Clin. Invest 129, 3909–3923. 10.1172/JCI125332 31424424 PMC6715358

[B40] Mason B. L. Wang Q. Zigman J. M. (2014). The central nervous system sites mediating the orexigenic actions of ghrelin. Annu. Rev. Physiol. 76, 519–533. 10.1146/annurev-physiol-021113-170310 24111557 PMC4019397

[B41] McKay N. J. Giorgianni N. R. Czajka K. E. Brzyski M. G. Lewandowski C. L. Hales M. L. (2021). Plasma levels of ghrelin and GLP-1, but not leptin or amylin, respond to a psychosocial stressor in women and men. Horm. Behav. 134, 105017. 10.1016/j.yhbeh.2021.105017 34174584

[B42] McLean C. P. Asnaani A. Litz B. T. Hofmann S. G. (2011). Gender differences in anxiety disorders: prevalence, course of illness, comorbidity and burden of illness. J. Psychiatr. Res. 45, 1027–1035. 10.1016/j.jpsychires.2011.03.006 21439576 PMC3135672

[B43] Menzies J. R. Skibicka K. P. Leng G. Dickson S. L. (2013). Ghrelin, reward and motivation. Endocr. Dev. 25, 101–111. 10.1159/000346058 23652396

[B44] M'Kadmi C. Cabral A. Barrile F. Giribaldi J. Cantel S. Damian M. (2019). N-terminal liver-expressed antimicrobial peptide 2 (LEAP2) region exhibits inverse agonist activity toward the ghrelin receptor. J. Med. Chem. 62, 965–973. 10.1021/acs.jmedchem.8b01644 30543423

[B45] Olff M. (2017). Sex and gender differences in post-traumatic stress disorder: an update. Eur. J. Psychotraumatol 8 (4), 1351204. 10.1080/20008198.2017.1351204

[B46] Patterson Z. R. Khazall R. Mackay H. Anisman H. Abizaid A. (2013). Central ghrelin signaling mediates the metabolic response of C57BL/6 male mice to chronic social defeat stress. Endocrinology 154, 1080–1091. 10.1210/en.2012-1834 23341196

[B47] Perello M. Cabral A. Cornejo M. P. De Francesco P. N. Fernandez G. Uriarte M. (2019). Brain accessibility delineates the central effects of circulating ghrelin. J. Neuroendocrinol. 31, e12677. 10.1111/jne.12677 30582239

[B48] Poe G. R. Foote S. Eschenko O. Johansen J. P. Bouret S. Aston-Jones G. (2020). Locus coeruleus: a new look at the blue spot. Nat. Rev. Neurosci. 21, 644–659. 10.1038/s41583-020-0360-9 32943779 PMC8991985

[B49] Roman C. W. Derkach V. A. Palmiter R. D. (2016). Genetically and functionally defined NTS to PBN brain circuits mediating anorexia. Nat. Commun. 7, 11905. 10.1038/ncomms11905 27301688 PMC4912612

[B50] Sahakian B. J. Winn P. Robbins T. W. Deeley R. J. Everitt B. J. Dunn L. T. (1983). Changes in body weight and food-related behaviour induced by destruction of the ventral or dorsal noradrenergic bundle in the rat. Neuroscience 10, 1405–1420. 10.1016/0306-4522(83)90122-7 6607427

[B51] Schalla M. A. Stengel A. (2019). LEAP2: a novel regulator of food intake and body weight? Nat. Rev. Gastroenterology Hepatology 16, 711–712. 10.1038/s41575-019-0224-9 31611666

[B52] Schéle E. Bake T. Rabasa C. Dickson S. L. (2016). Centrally administered ghrelin acutely influences food choice in rodents. PLoS One 11, e0149456. 10.1371/journal.pone.0149456 26925974 PMC4771210

[B53] Sciolino N. R. Hsiang M. Mazzone C. M. Wilson L. R. Plummer N. W. Amin J. (2022). Natural locus coeruleus dynamics during feeding. Sci. Adv. 8, eabn9134. 10.1126/sciadv.abn9134 35984878 PMC9390985

[B54] Skibicka K. P. Dickson S. L. (2011). Ghrelin and food reward: the story of potential underlying substrates. Peptides 32, 2265–2273. 10.1016/j.peptides.2011.05.016 21621573

[B55] Skibicka K. P. Hansson C. Alvarez-Crespo M. Friberg P. A. Dickson S. L. (2011). Ghrelin directly targets the ventral tegmental area to increase food motivation. Neuroscience 180, 129–137. 10.1016/j.neuroscience.2011.02.016 21335062

[B56] Skibicka K. P. Hansson C. Egecioglu E. Dickson S. L. (2012). Role of ghrelin in food reward: impact of ghrelin on sucrose self-administration and mesolimbic dopamine and acetylcholine receptor gene expression. Addict. Biol. 17, 95–107. 10.1111/j.1369-1600.2010.00294.x 21309956 PMC3298643

[B57] Smith A. Woodside B. Abizaid A. (2022). Ghrelin and the control of energy balance in females. Front. Endocrinol. (Lausanne) 13, 904754. 10.3389/fendo.2022.904754 35909536 PMC9334675

[B58] Toufexis D. Lipatova O. Johnson A. Abizaid A. (2016). Food‐restriction lowers the acoustic startle response in both male and female rats, and, in combination with acute ghrelin injection, abolishes the expression of fear‐potentiated startle in male rats. J. Neuroendocrinol. 28. 10.1111/jne.12436 27754564

[B59] Tschop M. Smiley D. L. Heiman M. L. (2000). Ghrelin induces adiposity in rodents. Nature 407, 908–913. 10.1038/35038090 11057670

[B60] Tufvesson-Alm M. Zhang Q. Aranäs C. Blid Sköldheden S. Edvardsson C. E. Jerlhag E. (2023). Decoding the influence of central LEAP2 on hedonic food intake and its association with dopaminergic reward pathways. bioRxiv, 555294. 2008.2029. 10.1101/2023.08.29.555294

[B61] Uriarte M. De Francesco P. N. Fernandez G. Cabral A. Castrogiovanni D. Lalonde T. (2019). Evidence supporting a role for the blood-cerebrospinal fluid barrier transporting circulating ghrelin into the brain. Mol. Neurobiol. 56, 4120–4134. 10.1007/s12035-018-1362-8 30276663

[B62] Van Bockstaele E. J. Reyes B. A. Valentino R. J. (2010). The locus coeruleus: a key nucleus where stress and opioids intersect to mediate vulnerability to opiate abuse. Brain Res. 1314, 162–174. 10.1016/j.brainres.2009.09.036 19765557 PMC3274960

[B63] Wald H. S. Ghidewon M. Y. Hayes M. R. Grill H. J. (2023). Hindbrain ghrelin and liver-expressed antimicrobial peptide 2, ligands for growth hormone secretagogue receptor, bidirectionally control food intake. Am. J. physiology. Regul. Integr. Comp. physiology 324, R547–R555. 10.1152/ajpregu.00232.2022 PMC10069974 36847494

[B64] Walf A. A. Frye C. A. (2007). The use of the elevated plus maze as an assay of anxiety-related behavior in rodents. Nat. Protoc. 2, 322–328. 10.1038/nprot.2007.44 17406592 PMC3623971

[B65] Walker D. L. Davis M. (1997). Anxiogenic effects of high illumination levels assessed with the acoustic startle response in rats. Biol. Psychiatry 42, 461–471. 10.1016/S0006-3223(96)00441-6 9285082

[B66] Wang J. H. Li H. Z. Shao X. X. Nie W. H. Liu Y. L. Xu Z. G. (2019). Identifying the binding mechanism of LEAP2 to receptor GHSR1a. Febs J. 286, 1332–1345. 10.1111/febs.14763 30666806

[B67] Wren A. Small C. J. Ward H. L. Murphy K. G. Dakin C. L. Taheri S. (2000). The novel hypothalamic peptide ghrelin stimulates food intake and growth hormone secretion. Endocrinology 141, 4325–4328. 10.1210/endo.141.11.7873 11089570

[B68] Yang B. Sanches-Padilla J. Kondapalli J. Morison S. L. Delpire E. Awatramani R. (2021). Locus coeruleus anchors a trisynaptic circuit controlling fear-induced suppression of feeding. Neuron 109, 823–838.e6. 10.1016/j.neuron.2020.12.023 33476548 PMC9272546

[B69] You Z.-B. Galaj E. Alén F. Wang B. Bi G. H. Moore A. R. (2022b). Involvement of the ghrelin system in the maintenance and reinstatement of cocaine-motivated behaviors: a role of adrenergic action at peripheral β1 receptors. Neuropsychopharmacology 47, 1449–1460. 10.1038/s41386-021-01249-2 34923576 PMC9206024

[B70] You Z.-B. Gardner E. L. Galaj E. Moore A. R. Buck T. Jordan C. J. (2022a). Involvement of the ghrelin system in the maintenance of oxycodone self-administration: converging evidence from endocrine, pharmacologic and transgenic approaches. Mol. Psychiatry 27, 2171–2181. 10.1038/s41380-022-01438-5 35064236 PMC9133122

[B71] Zigman J. M. Jones J. E. Lee C. E. Saper C. B. Elmquist J. K. (2006). Expression of ghrelin receptor mRNA in the rat and the mouse brain. J. Comp. Neurology 494, 528–548. 10.1002/cne.20823 PMC4524499 16320257

